# Attitudes and purchase intentions of polish university students towards food made from insects—A modelling approach

**DOI:** 10.1371/journal.pone.0300871

**Published:** 2024-03-29

**Authors:** Anna T. Mikulec, Anna M. Platta, Monika Radzymińska, Millena Ruszkowska, Karolina Mikulec, Grzegorz Suwała, Stanisław Kowalski, Przemysław Łukasz Kowalczewski, Marcin Nowicki

**Affiliations:** 1 Faculty of Engineering Sciences, University of Applied Science in Nowy Sącz, Nowy Sącz, Poland; 2 Faculty of Management and Quality Science, Gdynia Maritime University, Gdynia, Poland; 3 Faculty of Economic Sciences, Institute of Management Science and Quality, University of Warmia and Mazury in Olsztyn, Olsztyn, Poland; 4 Graduate of the Warsaw School of Economics, Warszawa, Poland; 5 Department of Food Product Quality, Krakow University of Economics, Kraków, Poland; 6 Faculty of Food Technology, Department of Carbohydrate Technology and Cereal Processing, University of Agriculture in Krakow Poland, Krakow, Poland; 7 Department of Food Technology of Plant Origin, Poznań University of Life Sciences, Poznań, Poland; 8 Department of Entomology and Plant Pathology, University of Tennessee, Knoxville, TN, United States of America; Central Research Institute of Electric Power Industry, JAPAN

## Abstract

The marketing of insect-derived protein has led to the development of respective legal regulations on such insects-based foods in the European Union. Despite the interest in the area of insect-based food, European researchers have paid relatively little attention to consumer attitudes and behaviors towards such products or the factors that may affect them. Attempts undertaken so far in this respect are insufficient; therefore, there is a need to continue and expand research in this field. The present study attempts to verify the following research hypotheses: H1. Attitudes towards food containing insects are related to the attributes/characteristics of these products, care for health and the natural environment, and attitudes towards novelty (neophilic/neophobic); H2. Intentions to purchase food containing insects can be predicted based on attitudes towards food from insects, product attributes, and attitudes towards environmental health and novelties. An empirical study was conducted among university students (N = 1063) by an indirect interview method using a specially designed questionnaire, via an online platform (Computer-Assisted Web Interview, CAWI) in November 2023. The questionnaire was validated by assessing the construction validity and estimating the reliability of the scales used. The study results demonstrated that the attributes of insect-based food products can influence the positive attitudes towards them and behavioral intentions to consume them, and that the strength of the impact of health quality traits is far greater than that of the organoleptic or functional traits. A negative, statistically significant value of the correlation coefficient between neophobic attitude and intention to purchase this type of food was observed. Thus, respondents without food neophobia were characterized by a positive attitude towards the purchase of foods containing edible insects in their composition.

## 1. Introduction

Foods and dishes made from insects are particularly valued by certain consumers in Asian countries, especially in parts of China, Thailand, and India, and also in parts of Latin America and Africa [1–13], where their consumption is not only based on traditions and dietary preferences but is also trendy. In turn, over the last few years, the European Union (EU) countries have expressed a growing interest in pursuit of alternative high-protein foods. This pursuit has led to the emergence of a new niche on the agri-food market and an increased interest in insect-derived protein. Production of insect protein can offer an attractive alternative to the costly production of animal protein, which is important due to the demographic explosion and the need to feed a growing human population. In addition, insects represent a source of excellent-quality feed for livestock [14] and a source of protein-, vitamin-, and mineral-rich human food [15,16]. The marketing of insect-derived protein has led to the development of respective legal regulations on the use of insects in the EU. Pursuant to Implementing Regulations (EU Commission) 2022/188 (of 10 February 2022) and 2023/5 (of 3 January 2023) [17,18], the EU Commission together with the European Food Safety Authority (EFSA) has approved the introduction of the frozen, dried, and powdered forms and partially skimmed powder made from house crickets (*Acheta domesticus*) into the consumer food market, towards the enrichment of confectionery, baked goods, beverages, and other food products, including corn meal-based snack products and snacks other than potato chips. The EU list of novel foods currently includes three types of insects: mealworm (*Tenebrio molitor*)—dried larvae; migratory locust (*Locusta migratoria*)—frozen, dried, and powdered forms; and the previously mentioned house cricket (*A*. *domesticus*)—frozen, dried, and powdered forms.

The available scientific literature highlights many benefits, including health, environmental, and economic ones, that stem from the production and consumption of insect-based food and dishes [19–23]. In addition, the insect food is indicated as the so-called sustainable food category, as it is an excellent source of protein, lipids, minerals and vitamins [24–26]. At the same time, it contains large amounts of fiber-like chitin polysaccharides, which can cause a feeling of satiety for a sufficiently long time and ensure prolonged time until the next meal [22,27–29]. Insects also contain antioxidative compounds [30], essential amino acids [31], and polyunsaturated fatty acids [32]. The ingredients of edible insects can improve gut health, exert anti-inflammatory and antioxidative effects, and reduce the risk of development of cardiovascular diseases. Vitamins and minerals found in insects contribute to proper mental development and bone health [33]. From the standpoint of sustainable development and environmental protection, breeding and processing insects is also associated with many environmental benefits as it, i.a., requires less area and smaller water consumption than conventional production, generates lower greenhouse gas emissions than mass production, and may use waste from the agri-food industry as inputs to produce food and feedstuffs, which further corresponds to the current trends of "zero waste" and "circular economy" [5,9,34–38]. Beside the environmental aspect, insect production ensures a higher feed conversion ratio compared to the conventional animal production [23,39–41]. The production of food containing insects is also economically viable. A literature overview has shown that the sales prices of insects are very diverse and depend on many factors, including: insect species, product type (larva, pupae, adult), forms of sales (fresh, processed–processing requires additional costs), type of market (sales prices of food products intended for human consumption are higher than for feed or pet food, which is due to higher quality requirements set for food products), and large differences in operating costs (e.g., water, electricity, labor, etc., and feed for insects) particularly between western and non-western countries where insect sales prices are relatively low [42,43]. The possibility of composing lower-cost diets due to the lower prices of insect protein than of the protein from conventional meat production is another economic benefit of insect food production [21,22]. However, it should be emphasized that this type of production requires establishing the precise cost estimates, including veterinary care, as well as generates concerns for food safety control and consumer acceptance assessment.

A review of the literature on insect food [44–56] has shown that the number of scientific publications in this field has increased dynamically over the last dozen or so years. In Europe, the subject matter has been undertaken by scientific and research centers in Belgium [44], Denmark [48,54], Germany [47,49], Portugal and Norway [53], and Italy [50]. The following areas of research interest can be distinguished within the works published in English-language literature that addresses food and dishes made from insects:

- the nutritional and feeding value of insect protein [57–59],
- the process of enriching food products with insect-derived protein [60–67],
- the motives of consumers when deciding to purchase food and dishes made from insects and assessing the degree of acceptance of products with added insects, e.g., bread [60], beef burgers and green lentils [44], jellies [7], protein energy bars [68], pasta [69,70], and oat cakes [71],
- the nutritional and environmental benefits and safety of insect consumption as a novel food source [72–76].

Despite the interest in the area of insect-based food, researchers have paid relatively little attention to consumer attitudes and behaviors towards these products and the factors that may affect them. Attempts undertaken so far in this respect should be deemed insufficient; therefore, there is a need to continue and expand research in this field. Many innovative projects and concepts related to the development of insect-based food products, including muffins, pâtés, corn extrudates, pancakes, sponge cake, and bars [23,69,77–81], have been elaborated, and their results are being implemented across Poland. Nevertheless, there is a cognitive gap in the Polish and central and eastern European scientific literature in this field. Undertaking research in this area is therefore justified, both from the standpoint of cognitive values and future implementation of the results.

This study is distinguished by the fact that it attempts to determine whether the attitudes of university students (young consumers) towards food containing insect protein and their declared intentions to purchase it will be related to the following variables (psycho-social determinants):

- concern for health,
- concern for the natural environment,
- attitude towards novelty (neophilic/neophobic)
- attributes/characteristics relevant to the purchase of novel, innovative food from insects.

The above variables were selected based on an overview of literature data of the subject. Authors of previous works have claimed that the health-promoting value [82,83] and ethical motives, including the environmental factors, play an increasingly important role in the decision-making process among consumers from developed countries. Persons who are concerned about the environmental degradation are more likely to accept novel types of food if they are convinced of the beneficial effects to the natural environment [73,84–87]. Factors influencing the consumer acceptance of insect-containing food, presented in the literature [51,88], included three categories: product attributes (e.g., price, quality, health benefits/risk, naturalness, and convenience of use); trust and social norms; and psychological factors (attitudes and culture). In addition, studies have demonstrated that food neophobia significantly and negatively affected consumers’ willingness to eat insect-based foods [89–91].

The present study attempts to verify the following research hypotheses:

H1. Attitudes towards food containing insects are related to the attributes/characteristics of these products, care for health and the natural environment, and attitudes towards novelty (neophilic/neophobic).
H2. Intentions to purchase food containing insects can be predicted based on attitudes towards food from insects, product attributes, and attitudes towards environmental health and novelties.

## 2. Research methodology

### 2.1. Subjects

The research results presented in this article derive from a questionnaire survey accomplished under an inter-university project conducted in five Polish entities offering higher education. An empirical study was conducted among university students, by indirect interview method using a custom-designed questionnaire, via an online platform (Computer-Assisted Web Interview, CAWI) in November 2023. Our study and the survey protocol received positive written consent from the University Ethics Committee for Research–Krakow University of Economics (KEBN/71/0044/D26/2023; 2023-10-27). Respondents gave written informed and voluntary consent to participate in the study and acknowledged the risk factors associated with participation in the CAWI study. The survey was conducted using the technique of non-probabilistic sample selection–purposeful sampling. Persons who were vegetarians, vegans, and on a flexitarian diet were excluded from the study. The study participants were persons who declared that they consumed all foods and did not limit their consumption of meat or animal products. During the research procedure, 1087 survey questionnaires were collected, and 24 incomplete and incorrectly completed ones were eliminated: 7 persons did not agree to participate in the study (they did not complete the survey further), 15 persons refused to answer question about gender, and 2 persons entered very large, unrealistic values in the age field. All respondents gave their free, informed consent to participate in the survey and were assured of its anonymity. The structure of the surveyed sample (N = 1063 respondents) is presented in Table 1.

**Table 1:** Characteristics of the study sample (N = 1063).

Variable	N	% of Total
**Gender**		
women	649	61.05
men	414	38.95
**Study profile**		
engineering-technical sciences	424	39.89
social sciences	382	35.94
medical and health sciences	121	11.38
exact and natural sciences	71	6.68
agricultural sciences	30	2.82
humanities	30	2.82
theological sciences	4	0.38
the arts	1	0.09
**Origin (Province)**		
Pomeranian	331	31.14
Lesser Poland	247	23.24
Warmian-Mazurian	182	17.12
Mazovian	63	5.93
Greater Poland	48	4.52
Subcarpathian	31	2.92
Kuyavian-Pomeranian	30	2.82
Podlaskie	27	2.54
Silesian	21	1.98
West Pomeranian	19	1.79
Lubuskie	18	1.69
Świętokrzyskie	12	1.13
Lublin	10	0.94
Lower Silesia	10	0.94
Łódź	9	0.85
Opole	5	0.47

Women accounted for ca. 61% of the surveyed population. The survey was conducted mainly among students of the following university profiles: engineering-technical sciences (ca. 40% of all respondents), social sciences (ca. 36%), medical and health sciences (ca. 11%), exact and natural sciences (ca. 7%); and being inhabitants of the following Provinces: Pomeranian (ca. 31% of all respondents), Lesser Poland (ca. 23%), Warmian-Mazurian (ca. 17%), and Mazovian (ca. 6%).

### 2.2. Questionnaire and data analysis

The research tool was constructed in such a way that the variables analyzed were a source of data allowing to achieve the assumed research goal and enabling verification of the research hypotheses under consideration. A set of statements adapted from studies/works by other authors was used while preparing the survey questionnaire (Table 2), which ultimately contained items related to:

- health attitudes—**HA** (5 items),
- environmental attitudes—**EA** (3 items),
- attitude towards novel food—**NF** (7 items),
- attributes of novel food: organoleptic traits **CA1** (3 items), health quality—**CA2** (3 items), functional traits—**CA3** (4 items),
- attitudes towards food made from insects—**ATT** (4 items),
- intention to purchase food made from insects—**PI** (5 items).

During the survey, the respondents expressed the level of approval or disapproval of all the items listed, using a 5-point Likert scale, where the values 1,2 meant: definitely no, no; value 3 denoted an answer: I do not know, I have no opinion; and values 4,5, corresponded to answers: yes, definitely yes.

**Table 2:** Variables and their measuring items.

Variables and their measuring items			Source
HA	1.1. The natural character of food products is an important quality attribute to me.		Kornher et al. (2019) [92]
	1.2. I try to buy organic food products.		
	1.3. I try to avoid food products containing food additives.		
	1.4. The quality certificate of purchased food is important to me.		
	1.5. The natural character of a production method is important to me.		
EA	2.1. When buying food, I try to pay attention to the fact how its production affects the natural environment.		Verbeke (2015) and Roberts (1996); Modlinska (2021) [84,91,93]
	2.2. I try to avoid food products, whose production has adverse effects on the natural environment.		
	2.3. I am interested in the impact of food production on the natural environment.		
NF	3.1. I am constantly trying new and different foods.		Pliner and Hobden (1992) [94]
	3.2 I do not trust new, unknown foods.		
	3.3 I do not try unknown foods.		
	3.4. I like foods from various national cuisines (ethnic food).		
	3.5. During parties/when I am out, I enjoy trying new foods.		
	3.6 I eat almost everything.		
	3.7. I like trying foods that are new to me.		
	3.8. The so-called "healthy food" looks too weird for me to eat.		
	3.9. I am afraid to eat something I have not eaten before.		
	3.10. I am very picky about the food I eat.		
CA1	4.1. Attractive taste.		Kornher et al. (2019) [92]
	4.2. Attractive aroma.		
	4.3. Attractive appearance.		

CA2	4.4. High nutritional value. 4.5. Health claims.		
	4.6. Nutritional claims.		

CA3	4.7. Various assortment and availability in retail. 4.8. Package size and attractiveness.		
	4.9. Convenience of use.		
	4.10. Availability of recipes on blogs and websites.		
ATT	5.1. I find buying novel food containing insects a good idea.		Wang et al. (2013) [95]
	5.2. I find buying novel food containing insects a wise choice.		
	5.3. I like the idea of buying novel, innovative food containing insects.		
	5.4. Buying novel, innovative food containing insects would be nice.		
PI	6.1. I would try dishes made from insects or with insect ingredients if I had the opportunity.		Kornher et al. (2019); Lee et al. (2010) [92,96]
	6.2. I am interested in eating dishes or food/food products made from insects in the near future.		
	6.3. If a "novel innovative food" appears on the market containing edible insects (fresh, frozen, dried, powdered, e.g., flour), which has such attributes as: reduction of CO_2_ emissions, nutritional claims, health claims, attractive taste, and high nutritional value I would be willing to buy it.		
	6.4. I am willing to buy new food containing edible insects.		
	6.5. I will make an effort to buy foods containing insect protein in the near future.		

Explanatory notes

HA—health attitudes; EA- environmental attitudes; NF—**-** attitude towards novel food; CA1—attributes of novel food—organoleptic traits; CA2—attributes of novel food—health quality; CA3—attributes of novel food—functional traits; ATT—attitudes towards food made from insects; PI- intention to purchase food made from insects.

The questionnaire was validated by assessing the construction validity and estimating the reliability of the scales used. The collected empirical material obtained from the research underwent statistical analysis using the following methods:

- **Exploratory factor analysis and scale reliability analysis based on Cronbach’s *α* coefficients.** These methods were used at the initial stage of data analysis to assess the validity and reliability of the scales used in the research tool.
- **Spearman correlation analysis**. This analysis was used to establish the strength and significance of correlations between dependent variables (ATT and PI) and independent variables (HA, EA, NF, CA1, CA2, and CA3).
- **Multiple Regression Analysis (MRA)** The MRA made it possible to build a model adjusted to empirical data, on the basis of which the potential of the examined independent variables (*X*) to explain consumer attitudes towards food made from insects and purchase intentions (*Y*), was estimated. The results were analyzed statistically using Statistica ver. 13.3.
- **Path analysis.** It was used to verify a hypothetical structural model depicting the immediate and intermediate effect(s) of the variables on the intention to purchase food made from insects. The latent variables of the model included, on the one hand, predictors of attitude (i.e., statistically significant variables selected on the basis of multidimensional linear regression analysis) and the attitude, and, on the other hand, the purchase intention. In-depth model verification was performed using R (ver. 4.1.2) and packages: lavaan (ver. 0.6.16) and tidySEM (ver. 0.2.4) (Bell Laboratories, New Jersey, USA). The criteria for assessing the quality of structural models are explicit. The quality of model fit was assessed using the RMSEA coefficient (*Root Mean Square Error of Approximation*), the Bentler CFI index (*Comparative Fit Index*), and the TLI index (*Tucker-Lewis Index*) [97,98].

## 3. Results

The validity and reliability of the measurement were estimated based on factor analysis and assessment of the internal consistency of individual scales using Cronbach’s *α* coefficients (Table 3). The reliability assessment method deployed is one of the most commonly used scale homogeneity measurement techniques [99,100]. The reliability analysis was based on the correlation coefficients of all items of a given scale with the overall score of the scale. The values of the Cronbach’s *α* coefficient range from 0 to 1. The minimal value of Cronbach’s *α* coefficient greater than 0.7 was adopted according to the Nunnally’s criterion [101,102]. Outliers, uncorrelated items and those that lowered the value of the reliability coefficient of a given scale were removed from the research tool. These included items 3.8, 3.9, and 3.10 of the NF scale. All scales used for further analyses were characterized by high internal consistency, above the threshold of satisfactory compliance (*α* > 0.7). Values of Cronbach’s *α* coefficients (Table 3) ranged from 0.82 to 0.94. The factor loadings of all items of the scale were above 0.6 (in the range from 0.65 to 0.97), meeting the criterion posited by Chin et al. [103].

**Table 3:** Measurement model: Reliability and validity.

Construct	Measurement item		Factor loadings	Cronbach α
HA	1.1.	-0.73		0.82
	1.2.	-0.78		
	1.3.	-0.74		
	1.4.	-0.75		
	1.5.	-0.82		
EA	2.1.	-0.92		0.86
	2.2.	-0.91		
	2.3.	-0.89		
NF	3.1.	-0.66		0.83
	3.2.	-0.65		
	3.3.	-0.66		
	3.4.	-0.65		
	3.5.	-0.77		
	3.6.	-0.71		
	3.7.	-0.82		
CA1	4.1.	-0.97		0.96
	4.2.	-0.97		
	4.3.	-0.96		
CA2	4.4.	-0.91		0.94
	4.5.	-0.96		
	4.6.	-0.97		
CA3	4.7.	-0.92		0.93
	4.8.	-0.88		
	4.9.	-0.93		
	4.10.	-0.88		
ATT	5.1.	-0.92		0.90
	5.2.	-0.95		
	5.3.	-0.91		
	5.4.	-0.76		
PI	6.1.	0.89		0.94
	6.2.	-0.93		
	6.3.	-0.95		
	6.4.	-0.86		
	6.5.	-0.83		

Abbreviations are defined in Table 2.

The degree of association between dependent variables and independent variables was determined in the next stage of the study (Table 4). Statistically significant correlations were found both between the variable "attitude towards food made from insects" (ATT) and the variables assumed in the model as well as between the variable "intention to purchase food from insects” (PI) and the examined variables, whereas the strength of these correlations was observed to vary. In both cases, the values of correlation coefficients indicated moderate and weak but statistically significant correlations (*p* < 0.05). The analysis of the ATT demonstrated it was the most strongly correlated with the variables related to the following attributes of novel food products: CA1 (*r* = 0.76), CA2 (*r* = 0.80), and CA3 (*r* = 0.77). In contrast, the variables "environmental attitude" EA (*r* = 0.22) and "health-promoting attitude" HA (*r* = 0.08) were weakly corrected with ATT. Nevertheless, these correlations were statistically significant (*p* < 0.05). In addition, a significant negative correlation (*r* = - 0.32) was observed between the variables "attitude towards novelty" NF and "attitude towards food made from insects" ATT. When considering the correlations between the PI and the other variables, the strongest statistically significant correlation (r = 0.80) was shown in the case of the variable ATT. There were also moderate statistically significant correlations between PI and CA1 (*r* = 0.59), CA2 (*r* = 0.75), and CA3 (*r* = 0.65). In turn, the variables HA, EA, and NF were weakly correlated with PI, with negative linear correlations found between NF and PI.

**Table 4:** Correlations between the attitude and the intention and the analyzed variables.

	HA	EA	NF	CA1	CA2	CA3	ATT
**ATT**	0.08*	0.22*	-0.32*	0.76*	0.80*	0.77*	
**PI**	0.12*	0.23*	-0.30*	0.59*	0.75*	0.65*	0.80*

Abbreviations are defined in Table 2; *statistically significant correlations (at *p* < 0.05).

In order to verify hypotheses H1 and H2, an attempt was made to examine the combined effect of independent variables on the dependent variables ATT and PI using multiple regression analysis (MRA). All statistically significant variables (*p* < 0.05) that correlated with the attitude (ATT) (Eq 1) and with the purchase intention (PI) (Eq 2) were included in the regression equation. The analyzed regression equations were as follows:

ATT=f(HA,EA,NF,CA1,CA2,CA3) (1)

PI=f(HA,EA,NF,CA1,CA2,CA3,ATT) (2)

In the next stage of the analysis, models were built and verified in accordance with generally accepted procedures. The results of the estimation of the model parameters enabled selecting independent variables which had a significant impact on ATT and PI. Tables 5 and 6 present the values of parameters related to the models indicating the variables important for the attitudes and the intentions of purchasing novel food from insects. The values of the coefficient of determination *R*^2^ of the proposed models were 0.69 and 0.70, which means that 69% and 70% of the total variability of the dependent variables ATT and PI, respectively, were explained by the adopted linear regression models. Thus, these models are characterized by a good fit to experimental data. According to the results obtained, the variable ATT (Table 5) was the most strongly influenced by the attributes of new products related to health quality CA2 (*β* = 0.41, *p* = 0.00) and organoleptic attributes CA1 (*β* = 0.33, *p* = 0.00), whereas the "functional trait" variable CA3 exerted a slightly weaker effect (*β* = 0.11, *p* = 0.01). In turn, the variables HA, EA and NF had the weakest influence on the variable ATT (*p*< 0.05). From the presented model showing variables important for the intention to purchase new food (Table 6), it appears that the strongest predictors of the variable PI were: attitudes toward food made from insects ATT (*β* = 0.64, *p* = 0.00) and health quality characteristics of the novel products CA2 (*β* = 0.59, *p* = 0.00). In turn, the variables CA1 and CA3 had a negative impact on PI, which was interpreted as reluctance of the persons paying much attention to organoleptic and functional attributes of food to purchase this food. But, the variables HA, EA and NF had no statistically significant (*p* < 0.05) effect on the model.

**Table 5:** Values of parameters related to the model indicating variables important for attitudes towards insects.

Variables	Non-standardized Coefficients *β*	Standard error	Standardized coefficients *β*	Values *t*	*p*
**Intercept term**	0.62	0.11		5.42	0.00
**HA**	-0.06	0.03	-0.05	-2.29	0.02
**EA**	0.08	0.02	0.07	3.47	0.00
**NF**	-0.09	0.03	-0.06	-3.49	0.00
**CA1**	0.28	0.03	0.33	10.89	0.00
**CA2**	0.37	0.04	0.41	9.66	0.00
**CA3**	0.09	0.04	0.11	2.47	0.01
***R*^2^ = 0.69 *F*(6.10) = 391.55**		standard error of the estimate = 0.72			
***p* < 0.00**					

Abbreviations are defined in Table 2.

**Table 6:** Values of parameters related to the model indicating variables important for the intention to purchase food made from insects.

Variables	Non-standardized coefficients *β*	Standard error	Standardized coefficients *β*	Values *t*	*p*
**Intercept term**	0.13	0.11		1.18	0.24
**HA**	0.02	0.02	0.02	0.78	0.44
**EA**	0.01	0.02	0.01	0.51	0.61
**NF**	-0.03	0.03	-0.02	-1.04	0.30
**CA1**	-0.16	0.03	-0.19	-5.99	0.00
**CA2**	0.51	0.04	0.59	13.50	0.00
**CA3**	-0.20	0.04	-0.23	-5.41	0.00
**ATT**	0.63	0.03	0.64	21.09	0.00
***R*^2^ = 0.70 *F*(7.10) = 352.04**		standard error of the estimate = 0.69			
***p* < 0.00**					

Abbreviations are defined in Table 2.

**Fig 1:**
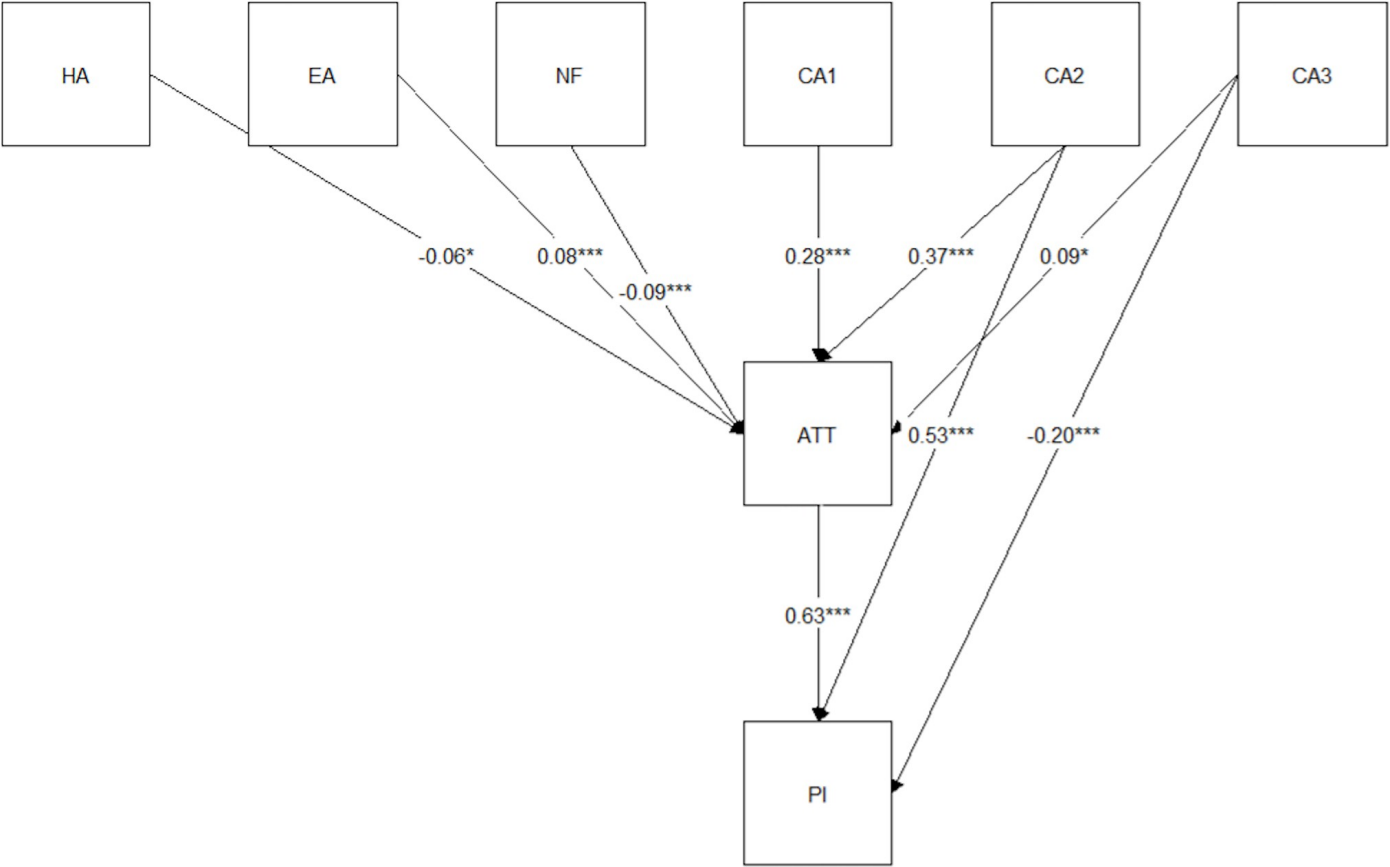
A structural model showing the intermediate and immediate impact of the predictors on the intention to purchase food from insects. **Explanatory notes:** Abbreviations are defined in Table 2; *values statistically significant at p<0.05; ***values statistically significant at p<0.001.

In the next stage of the study, an attempt was made to identify the variables which enabled predicting the intentions of purchasing food from insects. A path analysis was used to this end. The results of a multidimensional regression analysis showed that not all variables affected the intentions of purchasing food from insects. Only those variables that were statistically significant (*p* < 0.05) were included in the model. Fig 1 and Table 7 presents the parameters of the structural model accepted due to the matching measures of the structural model showing the intermediate and immediate impact of the variables on the intention to purchase food from insects. Good model fit was evidenced by the following indicators: RMSEA value not exceeding 0.06; the CFI = 1, which means that the model explains 100% of covariance; and TLI value level 1.

**Table 7:** Values of parameters related to the structural model.

Variables	Standardized β values	Standard error	Values	*p*
**ATT ~**				
**HA**	-0.06	0.02	-2.29	0.02
**EA**	0.08	0.02	3.48	0.00
**NF**	-0.09	0.02	-3.50	0.00
**CA1**	0.28	0.03	10.92	0.00
**CA2**	0.37	0.04	9.69	0.00
**CA3**	0.09	0.04	2.47	0.01
**PI ~**				
**ATT**	0.63	0.03	21.53	0.00
**CA1**	-0.16	0.03	-6.05	0.00
**CA2**	0.53	0.04	14.12	0.00
**CA3**	-0.20	0.04	-5.63	0.00

Abbreviations are defined in Table 2.

RMSEA = 0.00, Comparative Fit Index (CFI) = 1.00, Tucker-Lewis Index (TLI) = 1.00, *p* = 0.00, R2(ATT) = 0.69, R2(PI) = 0.69.

Our study suggested that the variable most strongly influencing the intentions of purchasing food from insects was related to the health quality of products CA2. That variable affected intentions both directly (*β* = 0.53, *p* = 0.00) and indirectly through attitudes ATT (*β* = 0.63, *p* = 0.00). Furthermore, it was found that the variables organoleptic attributes CA1 (*β* = -0.16, *p* = 0.00) and functional characteristics of the products CA3 (*β* = -0.20, *p* = 0.00) had an immediate negative effect on the intentions to purchase food from insects. In turn, these variables were found to affect indirectly, through attitudes ATT, the purchasing intentions PI at the levels: CA1 *β* = 0.28, *p* = 0.00 and CA3 *β* = 0.09, *p* < 0.05, respectively. The least dominant variables with the indirect (through ATT) influence on the variable PI were also HA, EA, and NF. These variables explained ca. 70% of the total variance (*R*^2^ = 0.69) for both ATT and PI (Fig 1). In addition, the study demonstrated strong correlations between ATT and PI (*β* = 0.63, *p* = 0.00). The proposed structural model offers a highly probable prediction of the intentions to purchase food from insects PI based on the variable “attitude toward food made from insects” ATT.

## 4. Discussion

Analysis of the results of empirical research presented in this manuscript enabled accomplishing the study goal and verifying the research hypotheses. The results of quantitative research allowed us to propose models indicating variables important for the attitudes and behaviors of young consumers towards food made from insects The models based on multidimensional linear regression analysis (Tables 5 and 6) and the path analysis (Fig 1), developed using empirical data, demonstrated that attitudes towards novel products as well as health and environmental attitudes do not play a significant role in explaining positive attitudes and intentions to purchase food from insects. The survey results proved that food quality characteristics (nutritional value, health claims, nutritional claims) were predictors of attitudes and behaviors of young consumers towards food made from insects. The study also showed that the intention to purchase such food can be predicted based on the assessment of the importance of sensory attributes and functional characteristics of these novel products. Thus, the research hypotheses (H1 and H2) advanced in the study were in part verified positively.

The paucity of data on attitudes and behaviors of young consumers towards insect food impairs in-depth comparison of the results obtained with other authors’ findings. In the light of the results obtained, it is also expected that those consumers who value the quality of products certified by the manufacturer will show a more positive attitude towards food from insects and the willingness to purchase it in the future. Other studies have confirmed that perception of beneficial features of a product, such as its health and nutritional value, may to some extent increase consumers’ willingness to try insect-based foods [49,51,88,104–110]. Some authors [111] have emphasized that sensory attributes of insect-based foods may be the dominant factors or barriers, depending on the degree of similarity to known foods. Processing insects into well-known food products promotes their acceptance. A growing interest has recently been noted in the subject of food neophobia in the acceptance of entomophages, especially in European countries where the concept of eating insects is relatively new [112–114]. One of the studies addressing this issue [115] demonstrated that persons with lower overall neophobia and a greater tendency to seek diversity tried food from insects earlier than those from the other studied groups. Negative effects of food neophobia on the acceptance of insects as food have also been observed [88] in both developed and developing countries, for example Italy [7,74,104,114–118], Germany [47,49,119–121], Poland [122], Switzerland [123], Australia [7], Hungary [46], Taiwan [124], China [106], and Uganda [125]. On the other hand, although the present study results demonstrated negative correlations between the level of neophobia and the attitudes and purchase intentions towards food from insects (Table 4), they failed to confirm that the neophobic attitudes among young consumers were important in explaining their positive attitudes and intentions to purchase food from insects. In view of the results obtained, it seems appropriate to further explore consumer attitudes toward novel foods. The proposed models, fitted to empirical data, are the starting point for further research. In the future, it would be necessary to verify the fit of the developed models with empirical data obtained from a broader range of subjects. In addition, the obtained study results confirm findings from earlier works proving that the attitudes make important prognostic factors of purchase intentions. The literature works emphasize the high prognostic usefulness of models illustrating the influence of attitudes on purchasing intentions of buyers [126–131].

Our findings could inform policymakers about potential strategies to promote the adoption of insect-based foods, such as incentivizing manufacturers to highlight health and environmental benefits on product labels or providing subsidies for research and development in this area. Furthermore, insights from the study can guide future marketing efforts to effectively communicate the nutritional and environmental benefits of insect-based foods, to target specific consumer segments identified as more receptive to such messaging. As such, understanding consumer preferences can inform product development efforts, guiding manufacturers in optimizing the sensory attributes and functional characteristics of insect-based food products to better align with consumer expectations. Indeed, educating consumers about the nutritional value, sustainability, and safety of insect-based foods through targeted educational campaigns could help overcome barriers to acceptance and foster more positive attitudes towards these products.

However, there are some limitations related to the presented study. The research was conducted in a narrow subjective approach and only among university students representing but a segment of young buyers. The proposed models, fitted to empirical data, generated the starting points for further research. In the future, they should be revised in a broader range of subjects. An additional follow-up longitudinal study could provide deeper insights into how attitudes and behaviors towards insect-based foods evolve over time. This would help understand the sustainability of acceptance and consumption patterns, allowing for more robust predictions and interventions and helping to predict market trends and to inform strategic planning.

## 5. Conclusions

We identified moderate to weak, but statistically significant, relationships between both attitude and the variables assumed in the model and intention and the variables studied. The study results demonstrated that the attributes of insect-based food products can influence the positive attitudes towards them and behavioral intentions to consume them, and that the strength of the impact of health quality traits is far greater than that of the organoleptic or functional traits. Intention to purchase food containing insects correlated most strongly with the purchasers’ attitudes towards insect food. Thus, respondents who did not exhibit food neophobia were characterized by positive attitudes towards purchasing foods containing edible insects in their composition. The variable that has the strongest effect on the purchase intentions for insect food is the characteristics related to the health quality of the products. This variable affects intentions both directly and indirectly through attitudes. From the proposed structural model, it is clear that purchase intentions for insect food can be predicted with high probability from the variable "attitudes towards insect food". As part of further research on consumer attitudes towards food made from insects in Poland, it is planned to: (a) characterize potential consumers of food from insects, and (b) identify factors that determine and diminish the demand for this category of food. Further research in this area seems useful and justified in order to support sustainable development in the environmental dimensions.
